# Duodenal major papilla morphology can affect biliary cannulation and complications during ERCP, an observational study

**DOI:** 10.1186/s12876-020-01455-0

**Published:** 2020-09-23

**Authors:** Po-Han Chen, Chun-Fang Tung, Yen-Chung Peng, Hong-Zen Yeh, Chi-Sen Chang, Chia-Chang Chen

**Affiliations:** 1 Division of Gastroenterology, Department of Internal Medicine, Taichung Veterans General Hospital, 1650, Taiwan Boulevard, Taichung, 40705 Taiwan; 2 School of Medicine, National Yang-Ming University, Taipei, Taiwan

**Keywords:** ERCP, Greater duodenal papilla, Endoscopic biliary Sphincterotomy, Endoscopy, gastrointestinal, Biliary tract surgical procedures

## Abstract

**Background:**

We investigated whether duodenal major papilla morphology could be a risk factor for failure of selective biliary cannulation (SBC) and post endoscopic retrograde cholangiography and pancreatography (ERCP) complications.

**Methods:**

A prospectively recorded database was reviewed retrospectively. Patients were included if they received therapeutic ERCP and had naïve major duodenal papilla. We used Haraldsson’s classification for papilla morphology, as follows: Regular (Type 1), Small (Type 2), Protruding or Pendulous (Type 3) and Creased or Ridged (Type 4). Risk factors for failing SBC and post-ERCP complications were analyzed by multivariate analysis.

**Results:**

A total of 286 cases were included. Age, gender, indications and therapeutic procedures were not different among the four types of papillae. The failure rates of SBC with Type 3 papilla and Type 4 papilla were 11.11% and 6.25%, respectively. In the multivariate analysis, Type 2 papilla (odd ratio 7.18, *p* = 0.045) and Type 3 papilla (odd ratio 7.44, *p* = 0.016) were associated with greater SBC failure compared with Type 1 papilla. Malignant obstruction compared to stone (odds ratio 4.45, *p* = 0.014) and age (odd ratio = 1.06, *p* = 0.010) were also risk factors for cannulation failure. Type 2 papilla was correlated with a higher rate of post-ERCP pancreatitis (20%, *p* = 0.020) compared to the other types of papilla However, papilla morphology was not a significant risk factor for any complications in the multivariate analysis.

**Conclusion:**

Small papilla and protruding or pendulous papilla are more difficult to cannulate compared to regular papilla. Small papilla is associated with a higher rate of post-ERCP pancreatitis.

## Background

Selective biliary cannulation (SBC) during endoscopic retrograde cholangiopancreatography (ERCP) is required in all therapeutic biliary procedures and is technically challenging. However, SBC can fail in up to 20% of cases even when performed by expert biliary endoscopists [1].

Multiple attempts at SBC increase the risk of post-ERCP pancreatitis (PEP), and other related complications [2]. The rates of complications related to attempting SBC range between 4 and 30% in the literature [2, 3].

Multiple factors are involved in biliary cannulation failure. For example, duodenal positioning, adequate visualization of the papilla, size of the papilla, variant patient anatomy, and surgery can affect the success rate in cannulation [4]. In biliary endoscopy, certain features of the papilla itself could influence cannulation difficulty. However, there are limited data on the association between papilla morphology and cannulation outcomes [5]. In 2017, Haraldsson et al. proposed four types of papilla. This classification is easy to use and has high inter- and intra-observer agreement [6]. Therefore, we started to register this papilla classification in our ERCP database.

In this study, we retrospectively reviewed our database and investigated whether there were any differences in cannulation failure rate and ERCP-related complications among the four types.

## Methods

### Data source

This study was conducted in Taichung Veterans General Hospital, a medical center with an annual ERCP volume of around 750–1000 (including naïve and non-naïve papilla). A prospectively recorded ERCP database was used in this study. Patients were included if they received therapeutic ERCP and had naïve major duodenal papilla. Diagnostic ERCP without any therapeutic intervention (e.g. biopsy, sampling, stenting, sphincterotomy, stone extraction) was few in our hospital and was not included in this study.

Exclusion criteria were age under 18 years old; pancreatic management (e.g., initial duct of interest was pancreatic duct); type 1 periampullary diverticulum (PAD), which often makes classification of papilla type and cannulation difficult); tumor involvement of the papilla; papilla could not be classified due to other reasons (e.g., duodenal mucosa swelling, deformity, ulcer on the surface of papilla); surgically altered anatomy or anomalous pancreaticobiliary ductal union, which makes cannulation difficult.

### Cannulation process

In our hospital, ERCPs were performed by experienced endoscopists who had performed over 1000 ERCPs. Trainees (fellows or young staff who had performed fewer than 1000 ERCPs) assisted with the ERCPs. If the lead endoscopists gave permission, the trainee attempted the initial bile duct cannulation. The lead endoscopists took over the cannulation if at any time during the procedure he felt the trainee was incapable of achieving deep bile duct cannulation. However, there was no minimal or maximal time limit for the cannulation performed by trainees. Likewise, no time limitation was imposed on trainees for cannulation attempts and pancreatic duct passage.

Sphincterotome and guidewire were used for biliary cannulation in the majority of cases. If the guidewire did not advance to the bile duct, minimal contrast injection was used to identify the distal duct anatomy, which thereby facilitated cannulation. If cannulation was still not successfully achieved, pre-cut with needle knife or transpancreatic sphincterectomy with/without pancreatic stent insertion was applied.

### Data collection in the database

**Fig. 1:**
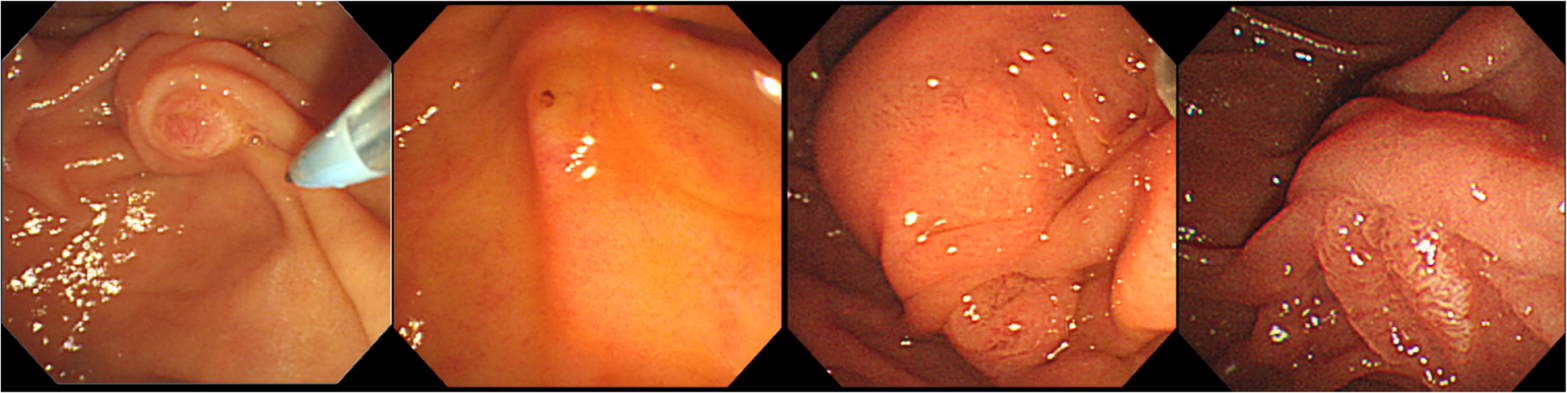
From left to right: Type 1; Regular papilla, with no distinctive features i.e. ‘classic appearance’. Type 2; Small papilla, often flat, with a diameter not bigger than 3 mm (approximately 9 Fr). Type 3; Protruding or pendulous papilla. A papilla is standing out, protruding or bulging into the duodenal lumen or sometimes hanging down, pendulous with the orifice oriented caudally. Type 4; Creased or ridged papilla, where the ductal mucosa seems to extend distally, rather out of the papillary orifice, either on a ridge or in a crease

Patients’ data related to the ERCP procedure were recorded including cannulation time (the time between first attempt of cannulation and secure placement of the guidewire in the common bile duct), the number of guidewire passages into the main pancreatic duct, cannulation success or failure, precut sphincterotomy, standard sphincterotomy, balloon sphincteroplasty, stone extraction, lithotripsy, endoscopic retrograde biliary drainage, and pancreatic stenting. Before cannulation, endoscopists classified the major papilla according to Haraldsson’s classification (Fig. 1): Regular (Type 1), Small (Type 2), Protruding or Pendulous (Type 3), and Creased or Ridged (Type 4) [6]. Then, bile duct cannulation was initiated. An endoscopic image was obtained when the endoscopist performed the first cannulation attempt (first intentional touch of the papilla). When a guidewire or catheter was securely placed inside the common bile duct, determined by fluoroscopy, another endoscopic image was captured. The total cannulation time could therefore be calculated. Passage of the guidewire into the main pancreatic duct and unintentional pancreatic contrast injection were recorded.

All ERCPs in our hospital were performed on an inpatient basis. After ERCP, all patients were followed up to assess immediate and delayed adverse events until discharge. The adverse events were recorded according to published guidelines [7]. Data on patients’ demographic data, underlying disease, indications for ERCP, renal function, liver function, platelet, and coagulopathy profile were also recorded.

### Interobserver agreement evaluation

In order to test our interobserver agreement, we collected all the endoscopic images of the ERCP included in this study. Individual image sets for each patient were reviewed for eligibility of interobserver evaluation. The eligible image sets must have all the following features: 1) good view of the papilla and the surrounding duodenal wall, 2) good close-up view of the papilla, 3) One frontal image with a standard sphincterotome positioned on the side of the papilla, acting as a yardstick. The eligible images sets were grouped according to individual patient. The images set were then sent to the endoscopists who had participated in this study. The endoscopists were asked to classify papilla type for each image set as they used to do. The endoscopists did not aware the previous classification of each image set. They also did not have other information about the patient’s characteristic and the procedure outcome. Interobserver agreement was calculated by comparing the classification of original database and the classification of the image sets in this interobserver agreement evaluation. The agreement was measured using kappa statistics.

### Investigating risk factors for cannulation failure and ERCP related complications

The aim of the study is to find risk factors for biliary cannulation failure and ERCP related complications. The investigated factors for biliary cannulation failure were patient’s demographic data, indications for ERCP, papilla type and laboratory data. The investigated factors for ERCP related complications were the factors listed above as well as variables related to the ERCP procedures.

SPSS (version 13.0; Chicago, IL, USA) was used for the statistical analyses. Categorical variables were compared by Chi-squared test and continuous variables were expressed as median and IQR, and compared by Mann–Whitney U test. Cox proportional hazard model was performed to analyze factors associated with cannulation failure and ERCP related complications, and significant factors (*P* < 0.05) in the univariate analysis were subjected to multivariate analysis to determine independent predictive factors. Statistical significance was defined as a *P* value of less than 0.05. The study was approved by the Ethical Review Board of TCVGH (CE19274B).

## Results

During October 2017 to October 2018, 316 therapeutic ERCPs involving naïve papilla were conducted at our hospital. Thirty cases were excluded for the following reasons. Five cases received pancreatic therapy (duct of interest was the pancreatic duct). Two papillae could not be found during the whole procedure, possibly because they were ectopic or obscure papillae. Three papillae were papilla tumors. Seven papillae were hidden in type 1 PAD. Five papillae could not be classified due to duodenal mucosa swelling, deformity, or ulcer on the surface of papilla. Seven cases had surgically altered anatomy. One case had anomalous pancreaticobiliary ductal union, which made cannulation difficult, and was thus excluded from the analysis. A total of 286 ERCPs were included in the final analysis. The Interobserver agreement was moderate to substantial (κ = 0.60, 95% confidence interval = 0.56–0.64) according our interobserver agreement evaluation. The demographic data of all patients are presented in Table 1. The mean age of the patients was 64 years old and 52.45% were male. The most common indications for ERCP were bile duct stones (73.08%), while 16.43% cases had malignancy-related bile duct obstruction. Among the malignant cases, 21 (44.68% of all 47 malignant obstructions) had bile duct cancer. The remaining 26 (55.32%) cases had non-biliary cancers, such as pancreatic cancer and metastatic lymphadenopathy.

**Table 1:** Demographic data of the patients

Total case	286
Age	64.00 ± 16.53
Papilla Type	
1	118 (41.26%)
2	25 (8.74%)
3	63 (22.03%)
4	80 (27.97%)
Gender	
Female	136 (47.55%)
Male	150 (52.45%)
Indications	
Stone	209 (73.08%)
Cancer	47 (16.43%)
Other	30 (10.49%)
Diabetes	76 (26.57%)
Hypertension	130 (45.45%)
CVA	19 (6.64%)
CRI	28 (9.79%)
COPD	9 (3.15%)
CAD	18 (6.29%)
Cirrhosis	14 (4.90%)
Pre-Cut	31 (10.84%)
Failed cannulation	17 (5.94%)
PAD	
0	166 (58.04%)
2	34 (11.89%)
3	86 (30.07%)

*CVA* Cerebral vascular disease, *CRI* Chronic renal insufficiency, *COPD* Chronic obstructive pulmonary disease, *CAD* Coronary artery disease, *PAD* Periampullary diverticulum

Most of the papillae were Type 1 (41.26%). Type 2 papilla was less common (8.74%). The overall cannulation success rate was 94.06%. In 31 patients (10.84%), precut was performed due to difficult cannulation. The complications rates were 0.7% for perforation, 6.64% for PEP, 4.2% for bleeding, and 1.75% for cholangitis. (Table 2).

**Table 2:** Complications of ERCP

Perforation	2	(0.70%)
Pancreatitis	19	(6.64%)
Bleeding	12	(4.20%)
Cholangitis	5	(1.75%)

Age and gender were not different in the four types of papillae (Table 3). The indications for ERCPs did not differ among the four papillae types. The patients with Type 1 papilla had the lowest prevalence of hypertension (36.44%). There were no significant differences in comorbidities, including diabetes, cardiovascular diseases, chronic renal diseases, chronic obstructive pulmonary disease, coronary artery disease, and cirrhosis among the different papilla types.

**Table 3:** Comparison between four papilla types

Papilla Type	1 (*n* = 118)	2 (*n* = 25)	3 (*n* = 63)	4 (*n* = 80)	*p* value
Age	63 (50.5–74)	65 (58–78)	68 (55–77)	67 (57.25–75)	0.213
Gender-Male	66 (55.93%)	11 (44.00%)	31 (49.21%)	42 (52.50%)	0.670
Indication					0.589
Stone	87 (73.73%)	16 (64.00%)	49 (77.78%)	57 (71.25%)	
Cancer	21 (17.80%)	4 (16.00%)	7 (11.11%)	15 (18.75%)	
Other	10 (8.47%)	5 (20.00%)	7 (11.11%)	8 (10.00%)	
Diabetes	27 (22.88%)	6 (24.00%)	21 (33.33%)	22 (27.50%)	0.490
Hypertension	43 (36.44%)	11 (44.00%)	26 (41.27%)	50 (62.50%)	0.003**
CVA	8 (6.78%)	2 (8.00%)	3 (4.76%)	6 (7.50%)	0.912
CRI	13 (11.02%)	2 (8.00%)	6 (9.52%)	7 (8.75%)	0.941
COPD	3 (2.54%)	0 (0.00%)	5 (7.94%)	1 (1.25%)	0.084
CAD	8 (6.78%)	2 (8.00%)	3 (4.76%)	5 (6.25%)	0.936
Cirrhosis	6 (5.08%)	2 (8.00%)	3 (4.76%)	3 (3.75%)	0.860
Pre-cut	8 (6.78%)	5 (20.00%)	10 (15.87%)	8 (10.00%)	0.117
Fail cannulation	2 (1.69%)	3 (12.00%)	7 (11.11%)	5 (6.25%)	0.037*
Pancreatic wire passage > 2	19 (16.10%)	8 (32.00%)	15 (23.81%)	22 (27.50%)	0.152
Pancreatic wire passage time	0 (0–1)	0 (0–1)	1 (0–2)	1 (0–3)	0.036*
Cannulation time					0.001**
0–5 min	67 (56.78%)	8 (32.00%)	19 (30.16%)	29 (36.25%)	
5–10 min	31 (26.27%)	4 (16.00%)	21 (33.33%)	23 (28.75%)	
> =10 min	20 (16.95%)	13 (52.00%)	23 (36.51%)	28 (35.00%)	
PAD Type					0.128
0	65 (55.08%)	13 (52.00%)	42 (66.67%)	46 (57.50%)	
2	12 (10.17%)	7 (28.00%)	5 (7.94%)	10 (12.50%)	
3	41 (34.75%)	5 (20.00%)	16 (25.40%)	24 (30.00%)	
Biopsy	14 (11.86%)	2 (8.00%)	8 (12.70%)	9 (11.25%)	0.938
Sphincterotomy	99 (83.90%)	22 (88.00%)	56 (88.89%)	72 (90.00%)	0.600
ERBD	39 (33.05%)	9 (36.00%)	15 (23.81%)	23 (28.75%)	0.540
ERPD	6 (5.08%)	2 (8.00%)	5 (7.94%)	5 (6.25%)	0.872
Stone extraction	75 (63.56%)	14 (56.00%)	41 (65.08%)	49 (61.25%)	0.864
Lithotripsy	2 (1.69%)	1 (4.00%)	2 (3.17%)	2 (2.50%)	0.880
EPBD	2 (1.69%)	3 (12.00%)	0 (0.00%)	1 (1.25%)	0.003**
Platelet	230 (169.75–297.75)	201 (155.5–310)	197 (146–276)	211 (152.5–281.75)	0.426
PT	10 (10–11)	10 (9.8–10.7)	10 (10–11.1)	10 (10–10.9)	0.502
APTT	28 (26.2–29.93)	27 (25.63–29.85)	28 (26.4–30.35)	28 (26.5–29.55)	0.961
AST	116 (47–227)	53 (22.5–150.25)	105 (46.75–205)	104 (42.5–238)	0.205
ALT	140 (48–312.5)	50 (21.5–125.5)	125 (55–265)	114 (44.5–309.75)	0.022*
Bilirubin	3 (1.38–6.13)	4 (0.45–5.1)	4 (1.5–6.1)	3 (1–5.08)	0.503
Creatinine	1 (0.67–0.97)	1 (0.56–1.27)	1 (0.66–1.06)	1 (0.68–1.02)	0.970
**Complications**					
Perforation	1 (0.85%)	1 (4.00%)	0 (0.00%)	0 (0.00%)	0.174
Pancreatitis	8 (6.78%)	5 (20.00%)	1 (1.59%)	5 (6.25%)	0.020*
Bleeding	6 (5.08%)	2 (8.00%)	1 (1.59%)	3 (3.75%)	0.525
Cholangitis	3 (2.54%)	0 (0.00%)	0 (0.00%)	2 (2.50%)	0.520

Kruskal-Wallis test. Chi-Square test. *p < 0.05, **p < 0.01

Continuous data were expressed as median and IQR

Categorical data were expressed as number and percentage

*CVA* Cerebral vascular disease, *CRI* Chronic renal insufficiency, *COPD* Chronic obstructive pulmonary disease, *CAD* Coronary artery disease, *ERBD* Endoscopic retrograde biliary drainage, *ERPD* Endoscopic retrograde pancreatic drainage, *EPBD* Endoscopic papilla balloon dilatation, *PT* Prothrombin Time, *APTT* Activated Partial Thromboplastin Time, *AST* Aspartate transaminase, *ALT* Alanine transaminase, *PAD* Periampullary diverticulum

Compared to other types of papillae, Type 1 papilla required significantly less time for cannulation (56.78% of the cases could be cannulated within 5 min) and had the lowest cannulation failure rate (1.69%). Type 2 papilla required more time for cannulation (52% of the cases needed at least 10 min) and had a cannulation failure rate of 12%. (Table 3) The failure rates for Type 3 papilla and Type 4 were 11.11 and 6.25%, respectively. (Table 3).

For the four types of papilla, there were similar percentages of therapeutic procedures performed, which included biopsy, sphincterotomy, biliary stenting, pancreatic stenting, stone extraction, and lithotripsy. However, endoscopic balloon papilla dilatation was used more often in the Type 2 papilla group (12%, *p* = 0.003).

The rates of complications, such as perforation, bleeding, and cholangitis, among the papilla types were similar (Table 3). Type 2 papilla had a higher percentage of PEP compared to the other types of papilla (Type 1, 6.78%; Type 2, 20.00%; Type 3, 1.59%; Type 4, 6.25%; *p* = 0.020). However, in the multivariate analysis, we did not find any risk factor (including papilla type) for any of the complications or for complications overall (data not shown).

The comparison between cannulation success and failure were shown in Table 4. There was significant difference in the papilla type and age between the success and failure group. (*p* = 0.037 and 0.010, respectively). There was also marginal difference in the indications for ERCP between the success and failure group. (*p* = 0.084) The risk factors for cannulation failure are shown in Table 5. In the univariate analysis, Type 2 or Type 3 papilla, cancer, and age were significant risk factors for cannulation failure. All of the above factors were also significant in the multivariable analysis. Cannulation was more difficult to perform for Type 2 papilla (odds ratio 7.18, *p* = 0.045) and Type 3 papilla (odd ratio 7.44, *p* = 0.016) than for Type 1 papilla. Malignancy-related obstruction was also a greater risk factor for cannulation failure compared to stone. (odds ratio 4.45, *p* = 0.014). Age was another risk factor for cannulation failure (odds ratio = 1.06, *p* = 0.010).

**Table 4:** Comparison between cannulation success and failure

Cannulation failure	No (*n* = 269)	Yes (*n* = 17)	*p* value
Papilla type			0.037*
1	116 (43.12%)	2 (11.76%)	
2	22 (8.18%)	3 (17.65%)	
3	56 (20.82%)	7 (41.18%)	
4	75 (27.88%)	5 (29.41%)	
Age	65 (53–75)	71 (65.5–85.5)	0.010*
Gender-Male	142 (52.79%)	8 (47.06%)	0.835
Indication			0.084
Stone	200 (74.35%)	9 (52.94%)	
Cancer	41 (15.24%)	6 (35.29%)	
Other	28 (10.41%)	2 (11.76%)	
Diabetes ^a^	74 (27.51%)	2 (11.76%)	0.255
Hypertension	123 (45.72%)	7 (41.18%)	0.909
CVA ^a^	16 (5.95%)	3 (17.65%)	0.093
CRI ^a^	27 (10.04%)	1 (5.88%)	1.000
COPD ^a^	7 (2.60%)	2 (11.76%)	0.094
CAD ^a^	17 (6.32%)	1 (5.88%)	1.000
Cirrhosis ^a^	14 (5.20%)	0 (0.00%)	1.000
PAD			
0	153 (56.88%)	13 (76.47%)	0.215
2	32 (11.90%)	2 (11.76%)	
3	84 (31.23%)	2 (11.76%)	
Platelet	215 (163–287)	171 (123–269)	0.159
PT	10 (10–10.9)	11 (10.15–11.65)	0.103
APTT	28 (26.1–29.7)	30 (27.4–31.6)	0.058
GOT	105 (41.75–220.75)	110 (50.25–170.25)	0.837
GPT	122 (47–290.75)	85 (29–185.5)	0.190
Bilirubin	3 (1.2–5.85)	3 (1.3–8.5)	0.754
Creatinine	1 (0.67–1.02)	1 (0.8–1.32)	0.100

Mann-Whitney U test. Chi-Square test. ^a^Fisher’s Exact test. *p < 0.05, **p < 0.01

Continuous data were expressed median and IQR

Categorical data were expressed number and percentage

*CVA* Cerebral vascular disease, *CRI* Chronic renal insufficiency, *COPD* Chronic obstructive pulmonary disease, *CAD* Coronary artery disease, *PT* Prothrombin Time, *APTT* Activated Partial Thromboplastin Time, *AST* Aspartate transaminase, *ALT* Alanine transaminase, *PAD* Periampullary diverticulum

**Table 5:** Risk factors for cannulation failure

	Univariate analysis		Multivariable analysis	
	OR (95% CI)	*p* value	OR (95% CI)	*p* value
Papilla				
1	ref.		ref.	
2	7.91 (1.25–50.12)	0.028*	7.18 (1.05–49.19)	0.045*
3	7.25 (1.46–36.04)	0.015*	7.44 (1.45–38.28)	0.016*
4	3.87 (0.73–20.44)	0.111	3.19 (0.58–17.43)	0.181
PAD				
0	ref.			
2	0.74 (0.16–3.42)	0.695		
3	0.28 (0.06–1.27)	0.099		
Indication				
Stone	ref.		ref.	
Cancer	3.25 (1.10–9.64)	0.033*	4.45 (1.35–14.66)	0.014*
Other	1.59 (0.33–7.72)	0.567	2.60 (0.47–14.23)	0.271
Diabetes	0.35 (0.08–1.57)	0.172		
Hypertension	0.83 (0.31–2.25)	0.715		
CVA	3.39 (0.88–13.01)	0.075		
CRI	0.56 (0.07–4.39)	0.581		
COPD	4.99 (0.95–26.13)	0.057		
CAD	0.93 (0.12–7.41)	0.943		
Cirrhosis	0.00 (0.00-	0.999		
Age	1.05 (1.01–1.09)	0.010*	1.06 (1.01–1.11)	0.010*
Gender-Male	0.79 (0.30–2.12)	0.647		
Platelet	1.00 (0.99–1.00)	0.297		
PT	1.29 (1.00–1.66)	0.054		
APTT	1.07 (0.99–1.16)	0.083		
AST	1.00 (1.00–1.00)	0.429		
ALT	1.00 (1.00–1.00)	0.346		
Bilirubin	1.03 (0.95–1.12)	0.469		
Creatinine	1.06 (0.59–1.89)	0.851		

Logistic regression. *p < 0.05, **p < 0.01

*CVA* Cerebral vascular disease, *CRI* Chronic renal insufficiency, *COPD* Chronic obstructive pulmonary disease, *CAD* Coronary artery disease, *PT* Prothrombin Time, *APTT* Activated Partial Thromboplastin Time, *AST* Aspartate transaminase, *ALT* Alanine transaminase, *PAD* Periampullary diverticulum

## Discussion

In this study, we used the endoscopic classification of papilla proposed by Haraldsson [6]. Small papilla (Type 2) and protruding or pendulous papilla (Type 3) were risk factors for failed SBC, compared with regular papilla (Type 1). In addition, cancer-related biliary obstruction and age were additional risk factors for SBC failure. Papilla types were not significant risk factors for any post-ERCP complications in the multivariate analysis. However, Type 2 papilla had a higher PEP frequency than the other types of papillae.

SBC is the first and most important step in ERCP. In the field of pancreatobiliary endoscopy, consistently achieving SBC is a milestone. The papilla itself can affect the difficulty of SBC. However, few studies have discussed the association between cannulation success and papilla morphology. In 2017, Haraldsson et al. proposed four types of papilla. This classification has substantial inter- and intra-observer agreement [6]. In another study, the authors found that SBC was more difficult for Type 2 and Type 3 papilla [8]. The definition of difficult cannulation that was used in the study was more than 5 min, 5 attempts, or 2 pancreatic guidewire passages, as per the Scandinavian Association of Digestive Endoscopy group study [9]. Our study had a similar finding. The percentages of cases with a cannulation time above 5 min for the four types papillae were 43.22% (Type 1), 68.00% (Type 2), 69.84% (Type 3), and 63.75% (Type 4). We also investigated the risk factors of cannulation failure, which was not discussed by Haraldsson et al. Other important risk factors for cannulation failure like age and malignancy related obstruction was not investigated in Haraldsson’s study. This is the most important difference between Haraldsson’s study and our study. In addition, cannulation failure means the patient needs another ERCP or alternative intervention for their disease, leading to increased medical cost and risk of complications. The biliary endoscopist should be especially cautious when attempting to cannulate Type 2 and Type 3 papillae.

Recently, another classification system of papillae was proposed by Watanabe et al. [10] Two important features of papilla were defined in this system. The first is the oral protrusion pattern, which indicates the ratio of the length of oral protrusion to the transverse diameter of the papilla, and it comprises three types: small (Protrusion-S), for which the ratio is less than one-half; regular (Protrusion-R), for which the ratio is one-half or more, but less than 2; and large (Protrusion-L), for which the ratio is 2 or more. They also identified Protrusion-L, which is very similar to Type 3 Papilla in the classification proposed by Haraldsson et al., as a significant risk factor for difficult biliary duct cannulation. The key feature of Type 3 Papilla and Protrusion-L papilla is large oral protrusion, with a longer biliary duct in the intramural distance. This may result in misalignment between the ERCP catheter and the biliary duct axis during biliary cannulation [10], thus leading to difficult cannulation and failure.

According to Watanabe et al., Protrusion-S is similar to the Type 2 papilla in Haraldsson’s classification. They found Protrusion-S was not difficult to cannulate. In contrast, Type 2 papilla was difficult to cannulate according to findings reported by Haraldsson and in the present study. This discrepancy in findings may be due to the fact that Protrusion-S was not exactly the same as Type 2 papilla. The defining feature of Type 2 Papilla is its small size. The small orifice of the papilla may explain why it is difficult to cannulate. It is often hard to insert the papillotome into the small biliary orifice. In contrast, the definition of Protrusion-S papilla is that it consists of a short oral protrusion. An oral protrusion that is short often means the intramural part of the bile duct is short, which is an obstacle for deep cannulation. It is often relatively easy to overcome a short intramural bile duct in Protrusion-S papilla.

We found age was a risk factor for cannulation failure. This result was similar to a finding reported by Emre et al. [11] They found the failure rate of cannulation had increased by 1.01-fold for each one-year increase in the patient’s age. Lobo et al. [12] found that cannulation success rates decreased significantly due to PAD as age increased. However, we found no significant relation between cannulation failure and PAD (we only included type 2 PAD and type 3 PAD). We excluded type 1 PAD because it made classification of papilla difficult. In our experience, large PAD type 1, which may obscure the papilla and distort its orientation, often makes SBC difficult. However, other age-related factors can cause cannulation difficulty. For example, duodenal distortion increases with age due to ulcers and cholangitis, which may make it difficult for operators to keep a good axis when approaching the papilla, thus resulting in difficult cannulation.

Fukatsu et al. [13] found malignant biliary stricture was a risk factor for needle-knife precut papillotomy. Freemann and Guda [3] also showed that malignant biliary tract obstructions decrease the cannulation success rate during ERCP. Our data also revealed malignancy-related biliary obstruction was a risk factor for cannulation failure. The cause of difficult cannulation in cancer patients may be due to tumor infiltration distorting and complicating endoscopic access to the ducts. Moreover, in patients with malignancy, papilla edema, trauma, and bleeding readily occur during ERCP because of fragile biliary tracts and vasculature, which thus makes cannulation more difficult [4].

### Complications

Our post-ERCP complication rates were similar to those of Haraldsson’s study [8]. Though we had not found any risk factors for post ERCP complications, the incidence rate of PEP was significantly higher for Type 2 papilla than for the other types. This finding is similar to that of Haraldsson’s study [8], i.e., small papillae were found to be associated with frequency of PEP, which increased in parallel with the frequency of difficult cannulation. We speculate that the cause of higher PEP rate in our Type 2 papilla was because endoscopic papilla balloon dilatation (EPBD) was used more often for this classification of papilla. In our cohort, the rate of EPBD for Type 2 papilla was 12%, which was much higher than that for the other types of papilla (*p* = 0.003). It is well known that EPBD with small-caliber (diameter = 8-10 mm) balloons increase PEP rate [14]. In our hospital, we preferred sphincterotomy over EPBD (diameter 8-10 mm was typically used) for most cases. But for some Type 2 papilla, we favored EPBD over sphincterotomy in order to reduce the risk of perforation. This preference was supported by a previous study that found small papilla was a risk factor for perforation associated with sphincterotomy [15].

### Limitation

This study had several limitations. First, the type of each papilla was classified by the endoscopist who performed that ERCP. There may be some disagreement among the endoscopists. However, our interobserver agreement evaluation shows moderate to substantial agreement, which is similar to that of Haraldsson’s study [6]. In addition, in our study, experienced endoscopists rather than fellows, determine the type of papilla. Therefore, we think the classifications of papilla in our study is reliable.

Second, biliary cannulation was often started by a fellow rather than an experienced specialist in our hospital. According to Haraldsson’s study, the rate of failed cannulation increased significantly when a fellow attempted the initial cannulation, even when an experienced endoscopist took over the biliary cannulation after 5 min [8]. Therefore, the rate of failed cannulation in our study might have been lower if experienced endoscopists had initially performed all biliary cannulations. However, we did not record the cannulation time of the fellows and experienced endoscopists, so it was not possible to estimate the influence of this variable on our study results. On the other hand, our rate of failed cannulation was only 5.94%. In previous report, SBC can fail in up to 20% of cases even when performed by expert biliary endoscopists [2]. We think the influence of trainee involvement was not so huge in our hospital. Further study is needed to evaluate whether Type 2 and Type 3 papilla are risk factors for failed cannulation when performed solely by expert endoscopists.

## Conclusion

We found small papilla (Type 2) and protruding or pendulous papilla (Type 3) were susceptible to failed biliary cannulation. Moreover, pancreaticobiliary cancer and advanced age were also found to be risk factors for biliary cannulation failure. Therefore, experienced endoscopists should be careful and consider taking over the cannulation process if trainees were involved in those cases.

## Supplementary information

**Additional file 1.** This is the data set used for investigating risk factors of cannulation failure and ERCP related complications.

**Additional file 2.** This is the data set used for interobserver agreement evaluation.

## Data Availability

We had provided the de-identified data supporting for this work in additional supporting files.

## Abbreviations

SBC: Selective biliary cannulation
ERCP: Endoscopic retrograde cholangiography and pancreatography
PEP: Post endoscopic retrograde cholangiography and pancreatography pancreatitis
PAD: Periampullary diverticulum
EPBD: Endoscopic papilla balloon dilatation
ERBD: Endoscopic retrograde biliary drainage
ERPD: Endoscopic retrograde pancreatic drainage
CVA: Cerebral vascular disease
CRI: Chronic renal insufficiency
COPD: Chronic obstructive pulmonary disease
CAD: Coronary artery disease
PT: Prothrombin Time
APTT: Activated Partial Thromboplastin Time
AST: Aspartate transaminase
ALT: Alanine transaminase
